# Modeling of Power Generation and Acid Recovery in an Analogous Process of Reverse Electrodialysis

**DOI:** 10.3390/membranes15040126

**Published:** 2025-04-20

**Authors:** Qiaolin Lang, Yang Liu, Gaojuan Guo, Fei Liu, Yang Zhang

**Affiliations:** 1 Shandong Engineering Research Centre for Pollution Control and Resource Valorization in Chemical Industry, College of Environment and Safety Engineering, Qingdao University of Science and Technology, 53 Zhengzhou Road, Qingdao 266042, China; langql@qibebt.ac.cn (Q.L.); liuyang@qust.edu.cn (Y.L.); 2 Qingdao Institute of Bioenergy and Bioprocess Technology, Chinese Academy of Sciences, 189 Songling Road, Qingdao 266101, China; 3 National Center for Materials Service Safety, University of Science and Technology Beijing, Beijing 100083, China

**Keywords:** reverse electrodialysis, acid gradient, acid recovery, power generation, modeling

## Abstract

The feasibility of an analogous reverse electrodialysis (RED) process for power generation and acid recovery from acidic waste streams in the steel industry is investigated in this study. A comprehensive model was established to simulate the transport phenomena and power generation, which was validated through experimental data. The simulated operation time was 3 h, during which an acid recovery rate of 41.7% was achieved, and the maximum output power density reached 30.37 μW·cm^−2^. The results demonstrated a strong dependence of output power density on the acid concentration, with a linear relationship within the tested range of 1.0–3.0 mol·L^−1^ HCl. An optimal flow rate range was identified that maximized power output, with the best value of 90 mL∙min^−1^. The differences in energy harvesting between the traditional acid diffusion dialysis process and our analogous RED process were demonstrated via simulation. The importance of system electroneutrality in driving ion migration and forming ionic currents was crucial for effective power generation. The analogous RED process is a promising solution for efficient acid recovery and power generation from industrial acid waste, offering a sustainable treatment approach.

## 1. Introduction

Investigating renewable energy is crucial for the sustainable development of our society [1,2]. Popular forms of renewable energy include solar, wind, tidal, geothermal, and bioenergy. Among these, solar and wind energy have been successfully applied at an industrial scale [3]. During the development of renewable energy, it has been discovered that significant chemical potential energy can be derived from salt concentration differences in natural and industrial processes [4]. Consequently, several commercial technologies have been developed to harvest salt concentration gradients, such as pressure-retarded osmosis (PRO) [5] and reverse electrodialysis (RED) in industrial application [6,7]. RED employs ion exchange membranes as its critical components, separating salty water from fresh water to maintain the salinity gradient [8]. The mechanism of RED involves alternately arranging cation exchange membranes (CEMs) and anion exchange membranes (AEMs) to facilitate the flow of anions and cations in opposite directions, forming an ionic current. The ionic current is then converted into an electronic current through the redox reactions of the electrode rinse solution.

The existing research on RED has primarily focused on optimizing membrane materials, stack configurations, and operating conditions to enhance power generation efficiency [9,10]. Previous studies have demonstrated that the performance of RED systems is significantly influenced by factors such as ion concentration gradients, flow rates, and membrane properties [11]. For instance, high salinity gradients generally lead to higher power outputs, while optimal flow rates are necessary to maintain efficient ion transport and minimize energy loss. Additionally, the use of highly efficient ion-exchange membranes plays a crucial role in maximizing the directed movement of ions and improving the overall system performance [12,13]. Our research group has recently conducted a systematic study on the impact of temperature on the power generation efficiency of RED, including the transport mechanisms of water and salt in RED [14]. Despite these advancements, there is still a need for further research to adapt RED technologies to specific industrial applications, such as the treatment of acidic waste streams, where unique challenges and opportunities arise [15,16].

RED mainly involves the use of salinity gradients to generate electricity. However, there is a significant amount of potential energy from acid gradients in industry. For instance, the global steel industry discharges a vast amount of acidic pickling wastewater every year [17,18], which contains substantial acid potential energy. In the traditional acid diffusion dialysis process, although acid purification and recovery can be achieved, the anions and cations migrate in the same direction; thus, no current can be formed. As a result, the potential energy from the acid gradient remains untapped and unconverted during the acid diffusion dialysis recovery process.

Aiming to fully utilize the acid gradient potential, Zhang Y. and Helson J. designed an analogous reverse electrodialysis system which utilizes acid diffusion membranes as the CEMs and proton blocking membranes as the AEMs [19]. Protons exclusively pass through the acid diffusion membrane at the acid concentration gradient between the acid chamber and the water chamber. Simultaneously, anions traverse the proton blocking membrane due to the system’s electric neutrality drive. Similar to RED, the ion current generated within the membrane stack is consequently converted into an electric current within the electrode compartments. Thus, during waste acid purification, the acid concentration gradient is simultaneously converted into electric power.

It is inevitable that sodium or chloride ions pass into the electrode chambers in RED, mixing with the electrode rinse solution. A similar situation also exists in this analogous RED system in generating power from the acid gradient. Typically, the cathode and anode chambers utilize potassium ferricyanide (K_3_[Fe(CN)_6_]) and potassium ferrocyanide (K_4_[Fe(CN)_6_]) solutions as redox agents to convert ionic currents into electronic currents. However, the presence of high concentrations of HCl in the feed solution poses a potential risk, as the reaction between HCl and cyanide ions can generate highly toxic hydrogen cyanide (HCN). In this way, the modeling method is popular for investigating power-generating processes in detail under diverse conditions, including in RED research. Two–dimensional multi–physical models for RED were established to investigate the effect of ion transport behavior on stack performance [20], stack module in serial configuration [21] and the coupling of RED with other processes [22]. The simulations offer valuable insights for concept validation and parameter optimization prior to experimental validation.

In this study, we aim to investigate the feasibility of using an analogous RED process for power generation and acid recovery from acidic waste streams generated in the steel industry. A comprehensive model was developed to simulate the transport phenomena and power generation involved in this process. The aim of this research is to provide insights into optimizing the system design and operating conditions for efficient energy recovery and acid recycling by understanding the relationship between acid concentration, flow rate, and power generation through simulation. The results of this study are expected to contribute to the development of sustainable solutions for treating industrial acidic waste streams, while also advancing the understanding and application of analogous RED technologies in specialized industrial waste acid.

## 2. Materials and Methods

### 2.1. Materials

Power generation and acid recovery experiments in an analogous process of reverse electrodialysis were performed to verify the modeling results. The solutions of 0.05 mol·L^−1^ K_3_[(Fe(CN)_6_] and K_4_[Fe(CN)_6_] and 0.25 mol·L^−1^ NaCl were prepared for the electrode rinse solution. K_3_[(Fe(CN)_6_], K_4_[Fe(CN)_6_] and NaCl were analytical-grade and supplied by Sinopharm Chemical Reagent Co., Ltd (Shanghai, China). An amount of 37 *wt*% HCl (Sinopharm Chemical Reagent Co., Ltd., Shanghai, China) was diluted to certain concentrations as feed solution for power generation testing. NaOH and phenolphthalein used in the acid recovery determination were purchased from Sinopharm Chemical Reagent Co., Ltd (Shanghai, China), both were analytical-grade. Commercial cation exchange membranes with proton-blocking capabilities (NEOSEPTA ACM) and anion exchange membranes with high-flux acid diffusion (NEOSEPTA AFN) were purchased from ASTOM Corporation (Tokyo, Japan), and were alternatively arranged similar to the stack configuration with RED.

### 2.2. Experiments

**Figure 1:**
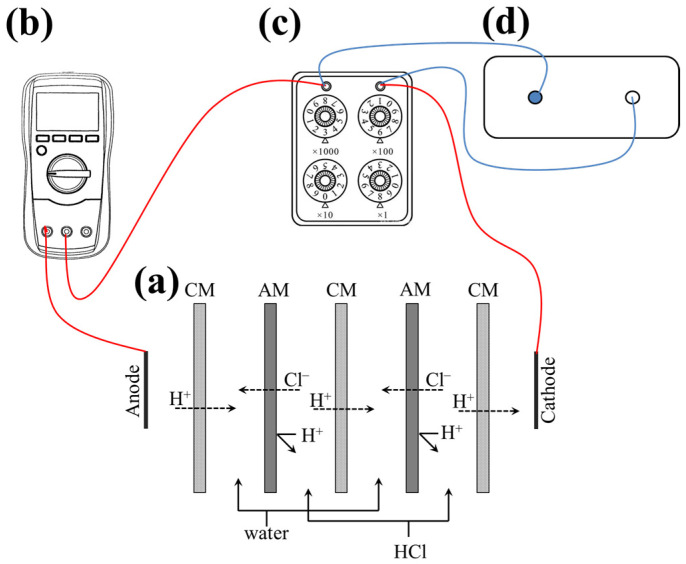
Scheme of analogous RED in power generation experiment: (**a**) membrane stacks of analogous RED, (**b**) digital multimeter for current measurement, (**c**) resistance box as the external load, (**d**) electrochemical workstation for voltage measurement.

The analogous process of reverse electrodialysis used in harvesting power from the acid gradient was facilitated in a membrane stack, which is shown in Figure 1. The main parts of the stack were alternatively arranged as the AM and CM stacks, by which compartments were divided into the feed and receive chambers. The acid was pumped into the feed chambers, while the water was introduced into the receive chambers. The flow rate of the feed and receive solution was optimized between 30 and 150 mL·min^−1^. At both ends of the stack, there were electrode chambers filled with the electrode rinse solution at a flow rate of 160 mL·min^−1^.

The output power of the acid gradient was determined using external load tests, as shown in Figure 1. With the stacks as the power source (Figure 1a), a six-decade resistance box in the range of 0–99.9999 kΩ (Figure 1c, ZX21, Shanghai Lichun Precision Instruments Co., Ltd., Shanghai, China) was used as an electronic load for the output power test. An electrochemical workstation (Figure 1d, CHI660E, CH Instruments, Shanghai, China) was used to measure the voltage drop across the membrane stack and load resistors. The current was measured with a 3½ digital multimeter (Figure 1b, UT890D, Uni-Trend Technology Limited, Guangzhou, China).

The determination of the acid recovery was based on the measurement of the acid concentration in the receive chamber. Samples were taken at regular intervals, and the H^+^ concentration was measured by titrating the diluted solution with sodium hydroxide, using phenolphthalein as the indicator.

### 2.3. Model Description

**Figure 2:**
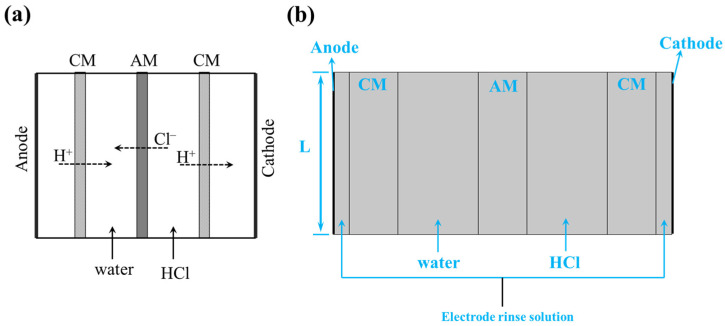
(**a**) Scheme of model in simulation, (**b**) geometrical set in simulation.

The simplified model consisted of electrodes, electrode chambers, feed and receive chambers, as well as the membranes (Figure 2a). Thus, the relative model in the COMSOL Multiphysics^®^ 6.1 [23] was established, as shown in Figure 2b. The height of the whole model was L, and the width of the electrode, receive, and feed chambers was 20 μm, 100 μm and 100 μm, respectively. The width of all the membranes was set to 60 μm. According to the stack configuration, the left channel of the AM was filled with water as the receive chamber, while the right channel of the AM was filled with 3.0 mol·L^−1^ HCl as the feed chamber. As shown in the scheme illustration of Figure 2a, the anionic ions transported through the AM from the feed to the receive chamber, forming anion ions flux from the right to the left side. The protons were transported through the CM from the feed to the next periodic receive chamber, forming cation flux from the left to the right side. Thus, the left side of the model should be the anode, while the right end should be the cathode.

### 2.4. Governing Equations

The laminar flow module and the tertiary current distribution module were used to solve the distribution of flux and the potential situation, respectively. The modeling of fluid in channels between two adjacent membranes takes the assumption of incompressible and laminar flows [20,24,25]. The governing equation for flux distribution is the Navier–Stokes equation [26], as follows:

(1) $$\rho \left(\frac{\partial u}{\partial t}+u\cdot \nabla u\right)=-\nabla p+\nabla \cdot \left(\mu \left(\nabla u+{\left(\nabla u\right)}^{T}\right)\right)+F$$

where u is the flow rate (m·s^−1^), ∇u describes the gradient of the flow rate, ρ is the density (kg·m^−3^), p is the hydrodynamic pressure (Pa), ∇p is the gradient of pressure, μ is the dynamic viscosity (Pa·s), and F refers to body forces (N·m^−3^). The incompressible continuity of Newtonian fluid is expressed as follows:

(2) $$\nabla \cdot \left(u\right)=0$$

which is solved simultaneously using Equation (1).

The governing equation for ion transport through the membrane obeys the Nernst–Plank equation [27,28], as follows:

(3) $${\vec{J}}_{i}=-D_{i}\nabla c_{i}-z_{i}u_{i}c_{i}F\nabla \phi_{l}+c_{i}\vec{u}$$

where ${\vec{J}}_{i}$ is the flux vector of ion *i*, $D_{i}$ is the diffusion coefficient of ion, $c_{i}$ is the concentration, $\nabla c_{i}$ represents the gradient of the concentration, $z_{i}$ is the charge number, $u_{i}$ is the mobility of ion *i*, *F* is the Faraday constant, ϕ is the electrical potential, $\nabla \phi_{l}$ is the gradient of the electrical field, the subscript *l* indicates the electrolyte phase, and $\vec{u}$ is the fluid velocity vector. The first, second, and third term in Equation (3) represent the diffusion flux, electromigration flux, and convection flux of ion i in the electrolyte, respectively.

The electrode kinetics at the cathode and anode surfaces was calculated using the Butler–Volmer equation [29,30]. The local current density *i*_ct_ at the electrode surface can be expressed as follows:

(4) $$i_{ct}=i_{0}\left\{C_{R}\left(\frac{\alpha_{a}F\eta }{RT}\right)-C_{O}\exp\left(\frac{-\alpha_{c}F\eta }{RT}\right)\right\}$$

where $i_{0}$ is the exchange current density, $C_{R}$ and $C_{O}$ are the dimensionless concentrations of the reducing and oxidizing substances, respectively, $\alpha_{a}$ and $\alpha_{c}$ are the anodic and cathodic transfer coefficients, η is the overpotential, R is the ideal gas constant, and T is the absolute temperature of the electrolyte. The first and second terms in Equation (4) represent the contributions of the anodic and cathodic currents at any electrode potential, respectively.

### 2.5. Boundary Conditions

The inlet boundaries were established at the lower edges of the feed and receive chambers, while the outlet boundary was defined at the upper edge. In the third current distribution of the electrochemistry module, the inflow and outflow settings were the same as those of the inlet and outlet, and contained the concentrations of ions. The value was initially specified as 0 for the feed chamber and 3.0 mol·L^−1^ for the feed chamber. The concentration in the feed chamber was also changed from 0 to 3.0 mol·L^−1^ to perform the parametric sweep study. In the simulation of the AM, the middle area in the geometry of the model was set as one type of separator, with the diffusion coefficient of protons of 0 m^2^·s^−1^ and the diffusion coefficient of chloride ions of 2 × 10^−9^ m^2^·s^−1^, thereby achieving the purpose of modeling a cation exchange membrane with proton-blocking performance. The electrodes at both ends of the model were set as the anode and cathode. The electrode reactions for anode and cathode were redox reactions between Fe^2+^ and Fe^3+^ ions. The current density of the electrolyte was also set as the boundary conditions for the simulation of the external load of resistance, in which the value of current density was set from 0 to 500 μA·cm^−2^ in a parametric sweep study to obtain the relative voltage. The voltage (V) of the load was calculated with the following equation:

(5) $$V(j)=OCV-R_{stack}\;j$$

where the OCV (V) is the open circuit voltage and *j* is the current density (μA·cm^−2^). The slope of voltage–current density curve represents the resistance *R*_stack_.

### 2.6. Data Processing

After obtaining the voltages at different current densities, the output power densities *P*(*j*) (μW·cm^−2^) were calculated with the following equation:

(6) P(j)=V×j

For the acid recovery, the recovery ratio (*R_acid_*) of acid was calculated to evaluate the acid recovery. The calculation formula is as follows:

(7) $$R_{acid}=\frac{c_{R}}{c_{F}}\times 100\%$$

where $c_{R}$ is the concentration of hydrogen ions in the receive chamber (mol·L^−1^), and $c_{F}$ is the concentration of hydrogen ions in the feed chamber (mol·L^−1^). Since the system is diffusion dialysis, the driving force for diffusion is the concentration difference. Therefore, the maximum theoretical recovery ratio is 50%.

## 3. Results and Discussion

### 3.1. Distribution of Flow Rate and Pressure

**Figure 3:**
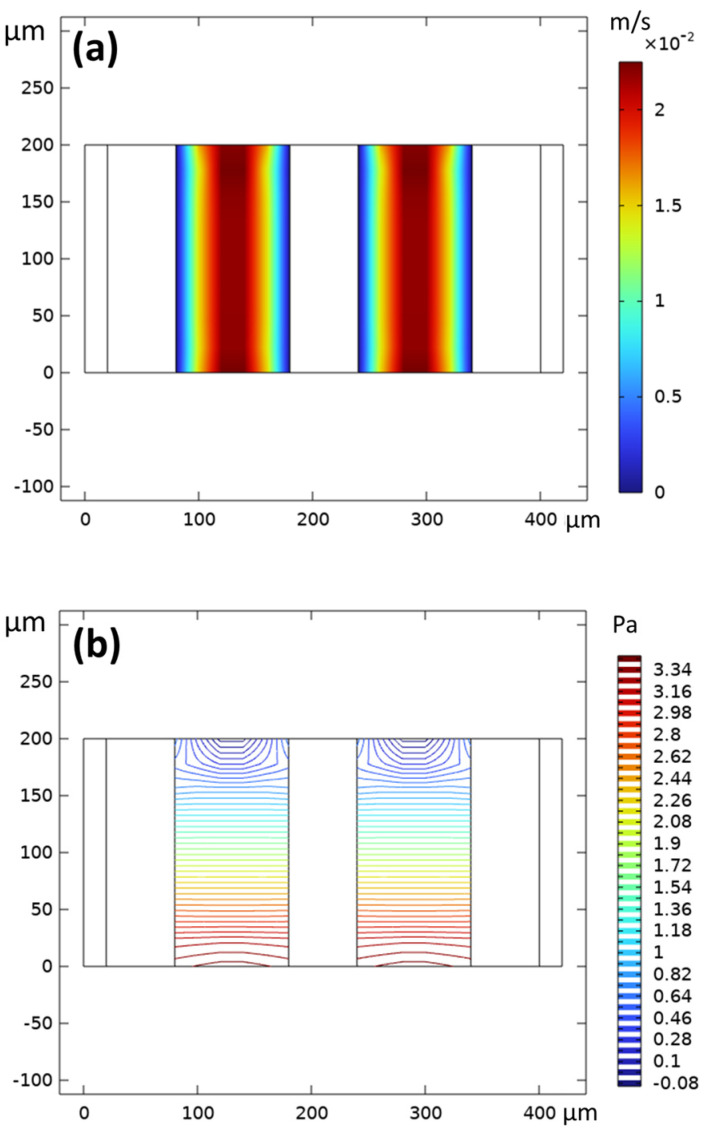
(**a**) Rate distribution (unit of the legend is m·s^−1^) and (**b**) pressure distribution of the liquid in channel (unit of the legend is Pa).

First, in the simulation of fluid, the velocity and pressure distribution of the fluid in channel was depicted at an initial inlet flow rate of 90 mL·min^−1^. As the influence of the spacer was neglected in the simulation, the flow within the channel was laminar with a boundary layer. As shown in Figure 3a, the velocity distribution conformed to the characteristics of laminar flow, with no-slip conditions at the walls. This indicated that the boundary conditions were appropriately set, which preliminarily suggested the successful establishment of the model. The pressure distribution revealed that the pressure was higher at the inlet and decreased towards the outlet. This pressure distribution (Figure 3b) was essentially symmetrical, which was consistent with the characteristic pressure distribution of fluid within the flow channels of an electrodialysis cell, while the pressure in the flow channel was lower than that of a conventional electrodialyzer. A possible reason for this was that the aspect ratio (width-to-length ratio) of the flow channel in the model was wider than that in a typical electrodialyzer. Additionally, a pressure drop existed along the entire flow channel, which is a common phenomenon, and can be attributed to the inherent fluid dynamic characteristics of the system.

### 3.2. Acid Concentrations in the Receive Chamber

**Figure 4:**
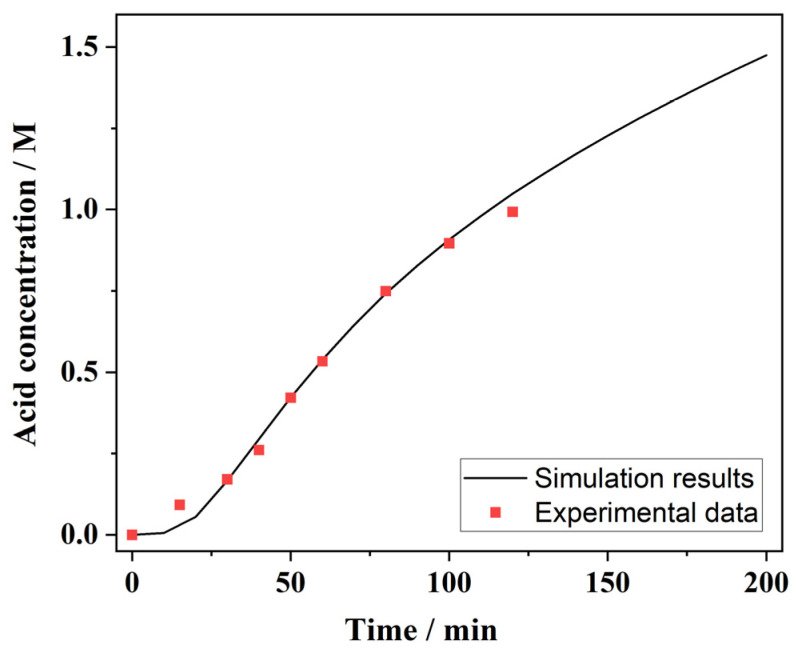
The simulation results (black curve) and experimental data (red dots) of the variation in the acid concentration with the running time in the receive chamber.

After confirming the normal performance of the fluid simulation, the diffusion dialysis settings were subsequently validated, with the recovery concentration of acid serving as the confirmation indicator. Initially, a probe was positioned near the outlet of the receive chamber to monitor the variation in the acid concentration during the dialysis process. According to the experimental results from our group’s prior work, the simulation runtime was set to 200 min, providing the temporal profile of the acid concentration as depicted in Figure 4 (red dots). The concentration increased with time and approached the theoretical value of 1.5 mol·L^−1^. This indicated that the model parameters and calculation conditions were reasonably set.

The acid concentration data obtained from the receive chamber under the same experimental conditions were compared with the simulation results (Figure 4, black curve). It can be seen that the experimental data were in good agreement with the simulation results, especially for the middle segment of the operating time. At the beginning of the operation, the experimentally measured acid concentration was slightly higher than the simulation results. A possible reason for this was that the simulation did not include the volume changes, whereas the experimental acid concentration was higher due to the initial volume decrease in the receive chamber. In the simulation results, the acid concentration was approximately 1.25 mol·L^−1^ when the operating time was around 3 h. According to Equation (7), the acid recovery rate was calculated to be 41.7%, which was close to the theoretical value of 50%. This indicated a relatively good acid recovery rate from the analogous process of reverse electrodialysis.

### 3.3. Output Voltages and Power Densities

**Figure 5:**
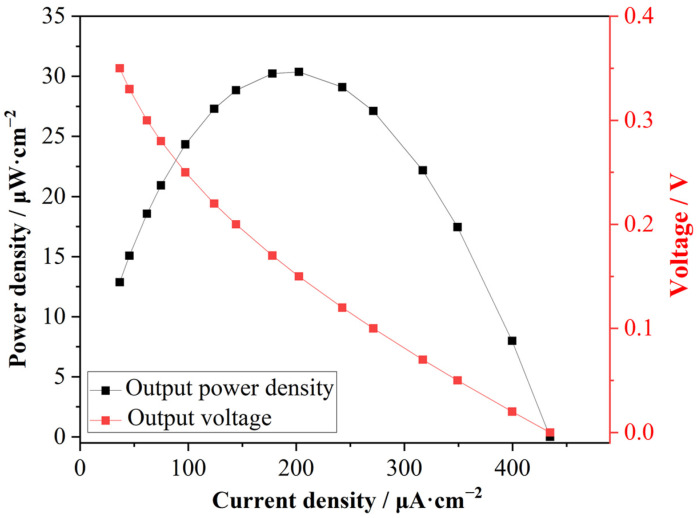
The output voltages (red dots) and power densities (black dots) at different current densities. Lines connecting the dots are for visual guidance only.

The simulation of power generation performance in this reverse electrodialysis-like system was then performed based on the modeling of the flow field distributions and the simulation outcomes from acid diffusion dialysis. This investigation employed an external circuit load approach, which was specifically realized through the implementation of a parametric sweep of current values in simulations. The polarization curve simulations were performed to obtain the relationship between the output voltage and current density, a methodology that is widely used in the power performance test of electrochemical fuel cells. As shown in Figure 5 (red dots), the output voltage *V* decreased with the increase in current density *j*, and the shape of the curve basically obeyed Equation (5) regarding the output voltage of the external load. The maximum current density was 434.57 μA·cm^−2^, corresponding to the output voltage of 0 V. The maximum output voltage was about 0.35 V, equal to the open circuit voltage (OCV). The power densities were calculated using Equation (6), and are reported in Figure 5 (black dots). The output power density curve was a typical parabolic shape, which was characteristic of power generation by battery-like components, and indicated that the simulation of power generation from the acid gradient was valid. The maximum output power density was 30.37 μW·cm^−2^, which was on the same order of magnitude as the power density of RED stacks.

### 3.4. Output Power Densities with Different Acid Concentrations

**Figure 6:**
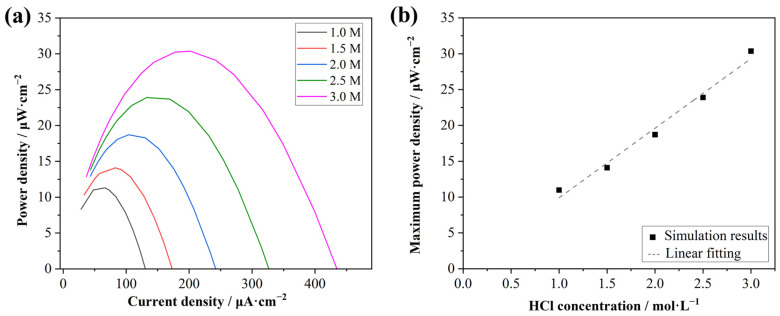
The simulation results of (**a**) power densities from different HCl concentrations (1.0, 1.5, 2.0, 2.5, and 3.0 mol·L^−1^) in the feed chamber, (**b**) the plot of power densities versus HCl concentrations (dots) and the linear fitting results (dashed line).

Investigating the influence of the acid concentration on power generation is of importance in the context of acid gradient power generation. Accordingly, in the simulation, different concentrations from 1.0 to 3.0 mol·L^−1^ of acids were employed as feedstock inputs to examine the variations in the power density output. The effect of the acid concentration on the output power was obvious, as shown in Figure 6a. The maximum current density and power density both decreased with the decrease in the acid concentration. A good linear relationship was presented between the maximum power density and HCl concentration, as shown in Figure 6b, with the calibration equation of *y* = 9.7132*x* + 0.1832 and the correction coefficient of 0.992.

In this simulation, the concentration range of acid was set to 1.0–3.0 mol·L^−1^, considering that the application scenario was the treatment of acidic waste streams generated in the steel industry, which typically contain 1.0–3.0 mol·L^−1^ HCl. Referring to the principles of RED, excessively high feed concentrations can lead to limitations in ion migration through the membrane, thereby causing a decline in the increasing trend of output power [31]. Conversely, insufficient feed concentrations result in an insufficient driving force for acid diffusion, which can lead to a significant decrease in the output power. The simulation results indicated that, within this concentration range, a good linear relationship existed between the acid concentration and the output power, thereby demonstrating that the design of the analogous RED process was well-suited for the intended application purpose.

### 3.5. Output Power Densities with Different Flow Rates

**Figure 7:**
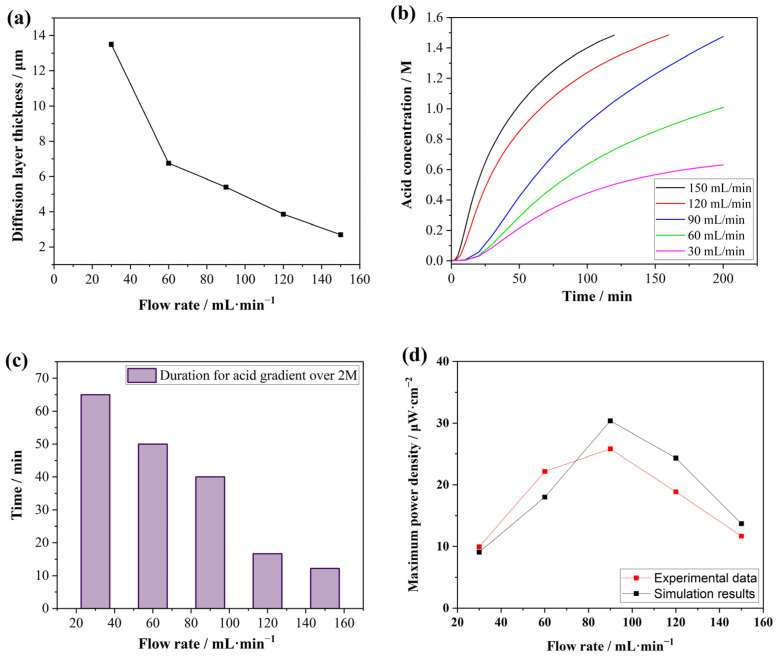
The simulation results of (**a**) diffusion layer thickness at different flow rates (dots), (**b**) the variation in the acid concentration in the receive chamber at different flow rates, (**c**) duration for acid gradient over 2 M at different flow rates, and (**d**) the power densities at different flow rates (black dots) and the comparison with the experimental data (red dots). In (**a**,**d**), lines connecting the dots are for visual guidance only.

Although ion transport through the membrane is perpendicular to the flow direction, the flow rate in the channel affects the diffusion layer at the membrane surface, thereby influencing the diffusion efficiency of ions as well as power generation. Therefore, simulations were conducted to investigate the output power density at different flow rates. The effect of flow rate on diffusion layer thickness is plotted in Figure 7a. It is clear that the diffusion layer thickness decreased at a higher flow rate, which led to the acceleration of ions passing through the membrane [32]. Thus, the output power was improved with the increase in the flow rate [33,34]. There were two factors of the flow rate effect for acid concentration in the receive chamber. First, the diffusion rate of acid increased with the increase in the flow rate, as shown in Figure 7b. Therefore, the output power was increased with the increase of the diffusion rate. The second effect of flow rate was that, since the system was set as a batch process, the acid gradient decreased more rapidly with the increase in the diffusion rate. Thus, the maintenance time when the acid gradient was greater than 2.0 M decreased rapidly at a higher flow rate, as shown in Figure 7c. The corresponding result was that the current density decreased more quickly, leading to a reduction in output power density.

For the above reasons, the output power density first increased and then decreased when the flow rate changed from 30 to 150 mL·min^−1^. The maximum value of the output power was obtained at a flow rate of 90 mL·min^−1^. This trend was consistent with the experimental results, indicating that the model’s settings for flow velocity-related parameters were reasonable. The simulated results were slightly higher than the experimental values, which was attributed to the absence of spacers in the model. Similar results in RED research reported that, at high flow rates, the diffusion boundary layer resistance dominates, while, at low flow rates, the resistance of the low-concentration solution becomes more significant [35]. They confirm that, in the medium flow rate range (30–70 mL·min^−1^), the power density remains relatively stable because increased ion diffusion in the low-concentration channel reduces the solution resistance, offsetting the increase in the diffusion boundary layer resistance [35]. The optimized flow rate of RED was smaller than that of the acid gradient power generation. A possible reason for this was that the thickness of the sodium ion’s diffusion boundary layer was greater than that of the proton, resulting in higher flow rates not being as effective for RED.

### 3.6. Comparison of Analogous RED with Diffusion Dialysis in Simulation

**Figure 8:**
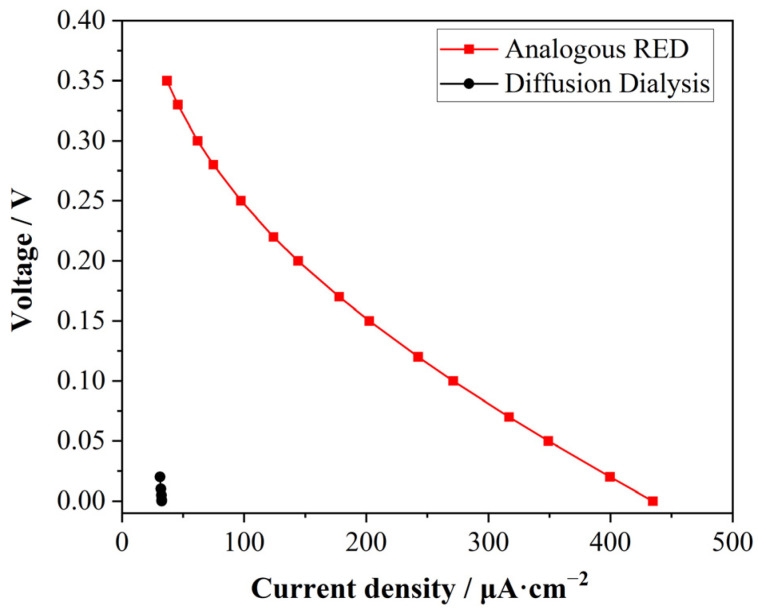
The output voltages at different current densities from analogous RED (red dots) and diffusion dialysis (black dots). Lines connecting the dots are for visual guidance only.

The difference in power generation between the general acid diffusion process and this analogous RED was simulated. To verify the key effect of the AM with the proton-blocking capacity in stacks for energy harvesting, the performance of the central AM was reconfigured to be the same as that of the CM. This led to the creation of a membrane stack generally used in acid diffusion which was entirely composed of acid diffusion-type CMs. The output voltage was then investigated. As shown in Figure 8, when the membrane stack configuration was altered to CM-CM-CM, the corresponding voltage for the output current density was significantly reduced. This indicated that, in the configuration composed entirely of diffusion dialysis membranes, the directed movement of ions was not established, and thus power generation could not be achieved. The simulation results verified the theoretical premise of acid gradient power generation. After replacing the central CM with the AM, the requirement of system electroneutrality forced ions to migrate directionally, forming an ionic current. The principle of electroneutrality decisively influences the behavior of ions, whether it is the migration of ions in solution or the interaction of ions with membranes [36,37]. Especially in the confinement theory, the electroneutrality of ions has an important impact on both microscopic and macroscopic behaviors [38,39].

### 3.7. Challenges in Industrial Application of Analogous RED

Although the analogous RED integrated the power generation of RED with the acid recovery of diffusion dialysis, it still encountered numerous challenges in industrial applications. The first and most evident issue is the corrosion of membranes and stacks caused by the acid. A potential solution to this problem is the use of durable membranes and corrosion-resistant electrodes. For instance, Iliev et al. explored the treatment of industrial liquid waste using RED, while simultaneously generating electrical power [7]. In their experiments, they use an electromembrane apparatus for acidic and alkaline waste treatment as the RED device [7]. In addition, AMs and CMs with acid resistance capacity were used in the analogous RED, which led to higher investment costs compared to standard RED. Therefore, the economic balance should be carefully considered when selecting the waste liquid for treatment. The waste acid, which was relatively expensive to treat, could potentially achieve economic benefits in industrial applications of the analogous RED process.

## 4. Conclusions

An analogous reverse electrodialysis system was successfully modeled. The power generation from the acid gradient was verified, and the acid was simultaneously recovered. The simulation results demonstrated that the acid recovery rate of 41.7% was achieved in a 3-h operation period, with a maximum output power density of 30.37 μW·cm^−2^. This study revealed that the output power density exhibited a strong dependence on the acid concentration, with a good linear relationship within the tested range of 1.0–3.0 mol·L^−1^ HCl. This indicates that the system design is well-suited for treating acidic waste streams from the steel industry. Additionally, the influence of flow rate on power generation was investigated, allowing for the determination of an optimal flow rate range that maximizes power output. The power generation ability of this analogous RED process compared with the general acid diffusion dialysis was also emphasized in the simulation. Overall, this research provided modeling insights for optimizing the analogous reverse electrodialysis process to ensure efficient acid recovery and power generation, offering a sustainable solution for industrial acid waste treatment. Future work may focus on further optimizing the system configuration and exploring the potential for scaling up the technology for practical industrial applications.

## Data Availability

The data that support the findings of this study are available from the corresponding author upon reasonable request.
